# Commentary: Cannabis use and resting state functional connectivity in the aging brain

**DOI:** 10.3389/fnagi.2023.1240511

**Published:** 2023-12-21

**Authors:** Cláudio Córdova, Otávio Toledo Nóbrega

**Affiliations:** Faculty of Medicine, University of Brasilia, Brasília, Brazil

**Keywords:** cannabis, *p*-value, Bayesian methods, statistical significance, clinical relevance

## Introduction

We would like to share ideas on the publication “Cannabis Use and Remaining Functional Connectivity State in the Aging Brain” that compares behavioral and neuroimaging data across users and non-users. As the primary outcome, Watson et al. (2022) identified greater connectivity of the hippocampus and of the parahippocampal cortex with targets in the anterior lobes of the cerebellum in older cannabis users compared to non-users. Despite the relevant results with promising applications in several clinical settings that enclose degenerative disorders as Alzheimer's or Parkinson's disease, Watson et al. interpreted their data relying on significance tests to make inferences. A *P*-value that surpasses an arbitrary level of statistical significance is not a guarantee that the effect produced by the intervention has practical implications or clinical relevance (Amrhein et al., 2019). Likewise, to reduce a significance threshold (α) or to have the *P*-value replaced by other statistics are not the solution either. *P*-values are just the tip of the iceberg (Leek and Peng, 2015; Amrhein et al., 2019). It is fundamental to go beyond a threshold of statistical significance and discuss the size of the observed effect (by including mean or median values, for instance), as well as the (im)precision of this estimate (by adding confidence intervals). Measures of central tendency and dispersion were not disclosed in the original paper, and we re-analyzed the main findings using the Bayesian method under the assumption that this approach can help interpretating results on a “more likely” or “less likely” basis.

## Reanalyses

The essential characteristic of Bayesian methods is their explicit use of probability for quantifying uncertainty in inferences based on statistical data analysis. Although one might assume that *P* < 0.05 is evidence against the null hypothesis (H_0_), this is not necessarily the case since *P*-values can be highly misleading measures. Being so, although Watson et al. have suggested significantly altered connections between the left hippocampus and the cerebellum (Vermis IV, V and Cerebellum III left) based on *P* = 0.049, analysis on *P*-values based on the Minimum Bayes factor [minBF(p)] suggests weak evidence for the refusal of *H*_0_. In other words, by converting *P*-value into minBF(p) = 0.40 (1/2.5), it could be concluded that the alternative hypothesis (*H*_1_) is only 2.5 times more likely of occurring than *H*_0_, being this level of evidence insufficient to allow assuming that the treatment (cannabis use) is effective to alter connections between targets, since the value lies under a score of 10 which is usually set as standard threshold (Stefan Angelika et al., 2019). Under the assumption of equal prior probabilities [P*r* (*H*_1_) = P*r* (*H*_0_*)* = 50%], the re-analysis found *H*_0_ with nonnegligible minimum posterior probability (MPP) of 28.7% (i.e., 1/3.484). If MPP of *H*_0_ is large, it would be appropriate not to reject the null hypothesis.

**Figure 1:**
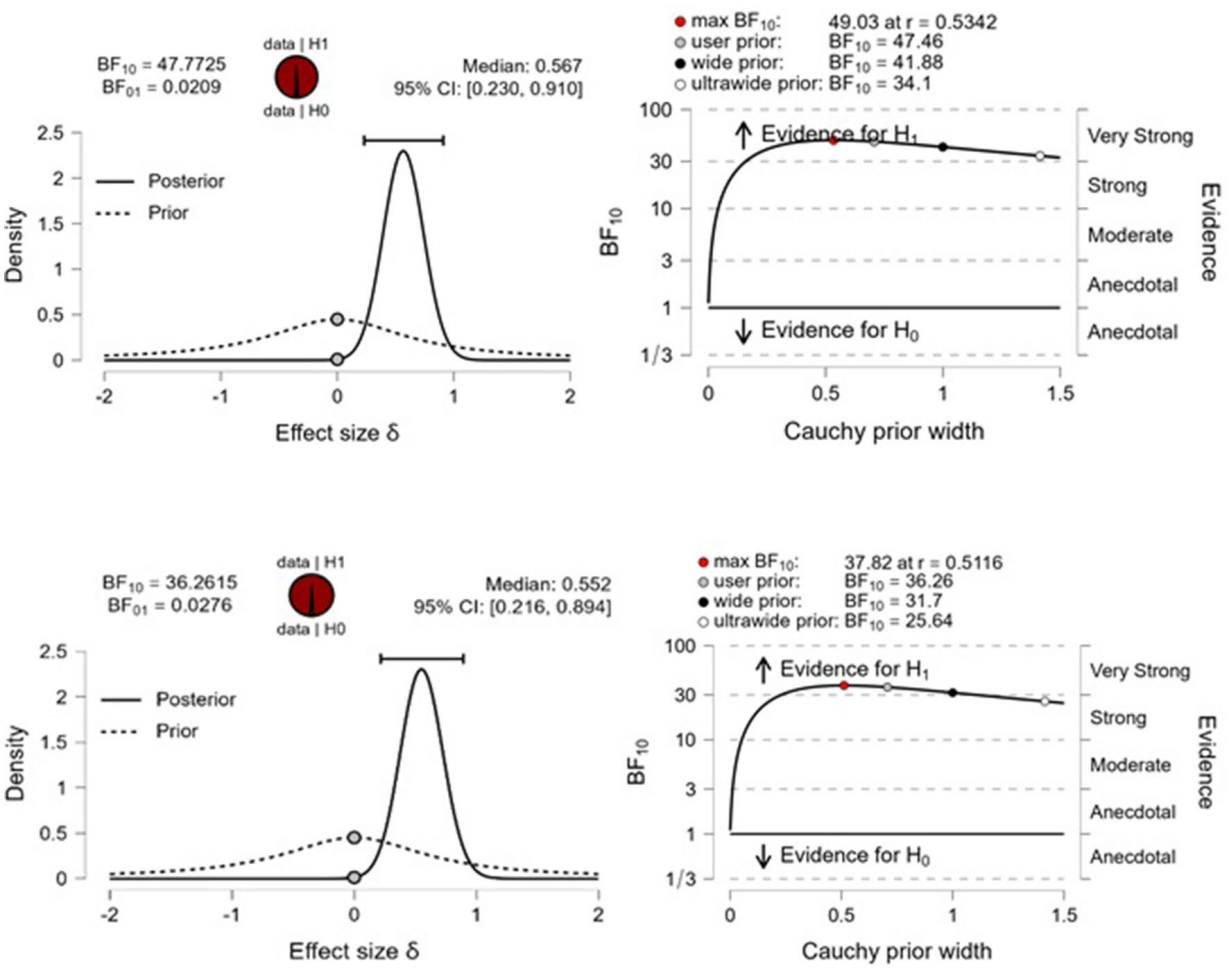
Bayesian two-sample *t*-test (Two-sided analysis for *H*_1_ estimation: δ Cauchy). **(Left)** The probability wheel on top visualizes the evidence in favor of the two rival hypotheses. The two gray dots indicate the prior and posterior density at the test value. The median and the 95% central credible interval of the posterior distribution are shown above the region with greater density. Figures on the **(right)** indicate the BF robustness plot. The maximum BF_10_ value is obtained when setting the prior width r ≈ 0.53. Analyses were conducted in Jasp 0.17.1 software.

On the other hand, a second re-analysis on data shown by Watson et al. has taken *T*-values (3.52 and 3.43) and group size (N_1_ = 43 and N_2_ = 143) into account. Through this method, the Bayes Factor (BF_10_) indicates probabilities in favor of *H*_1_ of the order of 48 to 36 greater in relation to H_0_, respectively (Figure 1). To examine the robustness of the Bayes factor (BF_10_), we used a wide range of prior distributions, as follows: user specific prior (*r* = 1/$\sqrt{2}$), wide prior (*r* = 1), and ultrawide prior (*r* = $\sqrt{2}$.) In this perspective, the evidence in favor of *H*_1_ is stable, suggesting that the analysis is robust. Most importantly, the *a posteriori* probabilities of 97.9% and 97.3% support a differential effect between elderly users and non-users, against the 2.1% and 2.7% chance that cannabis use does not change the connections at stake. In our re-analysis, the best estimate for an effect size was the median of 0.567 (Figure 1), with the credibility interval suggesting that the data is compatible with an effect of no < 0.230 or no more than 0.910 in magnitude.

Given the variability across re-analyses, the relationship between the *T* critical values and respective significance probabilities (as originally reported) was tested and revealed scores incompatible to each other (for *P* = 0.049, a *T* value of 1.98 is expected). For *T*-values of 3.52 and 3.43, our re-analyses suggest *P* ≤ 0.001. Under the circumstances described, we believe that the original authors have committed an error in some of their analyses or in the description of the results.

## Discussion

In summary, to better assess the value of a new therapeutic proposal, it is important to present sufficient information to answer the questions that motivated the research. One cannot properly conclude on potential benefits from a new therapy without knowing the estimated effect size of the treatment and the degree of statistical evidence. The level of confidence in experiments with “*P* < 0.05” cannot be the same for every significant result. In view of that, it is more informative to provide statistical conclusions that go beyond a single parameter and regardless of any preference in statistical approaches. In line, it is important that interpretating any probabilistic event should consider a solid theoretical foundation, aiming intervention in the context of clinical practices and the formulation of future actions or public health policies.

## Author contributions

CC: substantial contributions to study conception and design. ON: substantial contributions to acquisition of data. All authors contributed to the article and approved the submitted version.
